# Exploring stevioside binding affinity with various proteins and receptors actively involved in the signaling pathway and a future candidate for diabetic patients

**DOI:** 10.3389/fphar.2024.1377916

**Published:** 2024-08-07

**Authors:** Salman Khan, Nisar Ahmad, Hina Fazal, Ibrahim A. Saleh, Mostafa A. Abdel-Maksoud, Abdul Malik, Gehad AbdElgayed, Arshad Jalal, Kamran Rauf, Liaqat Ali, Sami Ullah, Niqabullah, Sajjad Ahmad

**Affiliations:** ^1^ Center for Biotechnology and Microbiology, University of Swat, Swat, Pakistan; ^2^ Pakistan Council of Scientific and Industrial Research (PCSIR) Laboratories Complex, Peshawar, Pakistan; ^3^ Faculty of Science, Zarqa University, Zarqa, Jordan; ^4^ Department of Botany and Microbiology, College of Science, King Saud University, Riyadh, Saudi Arabia; ^5^ Department of Pharmaceutics, College of Pharmacy, King Saud University, Riyadh, Saudi Arabia; ^6^ Integrated Molecular Plant Physiology Research, Department of Biology, University of Antwerp, Antwerp, Belgium; ^7^ São Paulo State University (UNESP), Ilha Solteira, Brazil; ^8^ Department of Horticulture the University of Agriculture Peshawar, Peshawar, Pakistan; ^9^ Department of General Medicine, Semey Medical University Kazakhstan, Semey, Kazakhstan

**Keywords:** diabetes, sugar alternative, stevioside, safe drug, insulin receptor substrate, molecular docking, Akt

## Abstract

**Introduction and Background:** Diabetes is a chronic metabolic disease characterized by elevated blood glucose levels and is one of the main global health concerns. Synthetic sugar substrate has many side effects such as leukemia, bladder cancer, hepatotoxicity, breast cancer, headache, and brain toxicity. The WHO and FDA has recently banned some of the synthetic sugar alternatives due to their carcinogenic effects.

**Objective and Methodology:** Therefore, the main objective of the current study was to investigate the safety and binding affinity of Stevioside with Glucose Transpoter-4 (GLUT-4), Akt, Insulin Receptor (IR) and Insulin Receptor Substrate-1 (IRS-1) to confirmed that Stevioside is one the potent natural sweetener/drug for diabetes. This study delves into the molecular interaction between Stevioside and key diabetic proteins: GLUT-4, Akt, IR and IRS-1. A precise molecular docking approach was used to simulate the binding affinity of Stevioside to these proteins. The pharmacokinetic properties of the molecule should be taken into consideration as important variables throughout the virtual screening process.

**Results:** The result of active site analysis of GLUT-4, Akt, IR and IRS-1 showed a zone of 2158.359 Ǻ^2^, 579.259 Ǻ^2^, 762.651 Ǻ^2^, and 152.167 Ǻ^2^ and a volume of 2765.094 Ǻ³, 355.567 Ǻ³, 686.806 Ǻ³, and 116.874 Ǻ³, respectively. Docking analysis of the Stevioside compound showed the highest docking energy with scores of −9.9 with GLUT-4, −6.7 with Akt, −8.0 with IR and −8.8 with IRS-1. Studies indicated that it remains undigested by stomach acids and enzymes and is not absorbed in the upper small intestine. Further, tests revealed no hepatotoxicity, AMES toxicity, or skin sensitivity, making it a promising candidate for safe consumption as drug metabolism.

**Conclusion and Recommendations:** Instead of other sugar alternatives, Stevioside will help diabetic patients with a lower chance of infections, lowered blood pressure/blood sugar, and increased glucose uptake in diabetic muscles. Stevioside is a natural sweetener, and the current study recommends its usage in various dietary products for diabetic patients.

## 1 Introduction

Diabetes mellitus is a chronic metabolic condition caused by high blood glucose levels that directly or indirectly affect the heart, blood vessels, eyes, kidneys, and nervous system over the time (Jwad and Al-Fatlawi, 2022). Diabetes is categorized as type 1 (T1D) and type 2 (T2D). When the immune system of the body targets and damages pancreatic beta, this is known as T1D, while when the body’s cells do not respond normally to insulin, this is known as T2D (Szablewski, 2014). T2D is a multifactorial disease caused by a combination of genetic, lifestyle, family history, age, environmental factors, physical inactivity, and certain medical conditions like high cholesterol and high blood pressure (Egede and Dagogo-Jack, 2005). T2D resists and impairs the production of insulin, which leads to higher blood sugar levels by disrupting the function of the pancreas to maintain optimal sugar in the blood (Masharani and German, 2011). Symptoms of T2D may not be apparent initially but can include increased thirst, frequent urination, fatigue, and blurred vision, as well as other serious diseases (Balaji et al., 2019). To control diabetes, patients use different artificial sweeteners, such as saccharin, aspartame (AS), acesulfame-K (Ace-K), and sucralose (Fowler et al., 2008). However, artificial sweeteners like saccharin have been found to cause cancer in rats and dogs, while causing allergic reactions such as headaches, skin problems, and diarrhea in humans (Tuormaa, 1994; Oo, 2019). Hereby, some countries, like Canada and the United States, banned the use of saccharin against diabetes (Shelat et al., 2020). In addition, acesulfame k (Ace-K) and sucralose also caused a variety of health problems, such as the production of the toxic substance “aceto-acetamide,” eye irritation in animals, shrunken thymus gland (up to 40%), decreased red blood cell count, enlarged liver, and kidney diseases (Belton et al., 2020). Aspartame (AS) has been used as a novel sweetener in the United States since 1974; however, several studies indicated that AS may cause brain toxicity and cancer (Landrigan and Straif, 2021). Therefore, natural sweeteners are considered the best alternative sugar substitutes that provide a sweet taste.

Natural sweeteners are extracted from plants with less or no side effects and can be used in the pharmaceutical and food industries (Shelat et al., 2020). Some of the potential natural sweeteners including *Glycyrrhiza glabra* and *Stevia rebaudiana* gained the attention of the global community due to their safe consumption by diabetic patients (Putnik et al., 2020; Saraiva et al., 2020).

Modern biotechnology offers a powerful arsenal for engineering and optimizing SG biosynthesis pathways in *S. rebaudiana*. This includes a comprehensive suite of techniques encompassing metabolomics, proteomics, and transcriptomics (Bayraktar et al., 2018). These approaches enable researchers to gain a deep understanding of the metabolic processes of plants at the molecular level. By manipulating these pathways, scientists can potentially improve the overall agronomic performance of stevia, including aspects like genome architecture, morphology, and physiological characteristics (Philippe et al., 2014). Additionally, specific techniques like micropropagation offer a robust platform for *in vitro* propagation of stevia plants, facilitating large-scale production of plant biomass and the targeted accumulation of medicinally or biologically active compounds like SGs (Basharat et al., 2021). An *in vivo* study by Modi and Kumar (2018) investigated the effects of exogenous gibberellic acid (GA) on stevioside accumulation in *S. rebaudiana* plants. Treatment with various GA concentrations (15, 30, and 60 μM) resulted in a more than two-fold increase in stevioside content compared to the control group. This suggests the potential of plant growth regulators (PGRs) for enhancing SG biosynthesis *in vivo* (Modi & Kumar, 2018). Further research by other authors explored the impact of methyl jasmonate (MeJA) and elicitors on stevioside levels. Plants treated with varying concentrations (50, 100, 150, and 200 μM) of MeJA and elicitors exhibited increase stevioside content, with the highest concentration (200 μM), leading to an increase from 8.14% (control) to 10.33%. Interestingly, these studies revealed different expression patterns in genes associated with the stevioside biosynthesis pathway under these treatments, suggesting complex regulatory mechanisms at play (Dezhsetan et al., 2017; Modi & Kumar, 2018). The influence of certain biofertilizers, including vesicular-arbuscular mycorrhiza (VAM) fungi, *Azospirillum* bacteria (AZO), and phosphorus-solubilizing bacteria (PSB), on stevioside production and their enhancement have also been investigated (Kuntal Das and Raman Dang, 2010; Vafadar et al., 2014). Furthermore, biotechnology and biopharmaceutical industries suggested that the market value of stevioside was $338 million in 2015 and was projected to reach $554 million by 2024 by a six-fold increase (MVM, 2020).

Stevia is a sweet-leaf semi-humid subtropical plant in the Chrysanthemum family (Asteraceae), which is 100–300 times sweeter than sucrose. Stevia contains sweet compounds such as glycosides, which include stevioside and rebaudioside (Abbas Momtazi-Borojeni et al., 2017). Stevioside is a kind of glycoside that has glycosyl residues bound to its cyclopentanoperhydrophenanthrene skeleton (Shelat et al., 2020). Stevia does not affect blood glucose levels, which may be good for diabetes. Stevioside can help in weight control and obesity management (Ashwell, 2015). It may also have potential in cancer prevention by targeting breast cancer cells without inducing allergic reactions (Iatridis et al., 2022). In addition, stevia has antifungal and antibacterial properties and is also deemed safe for diabetics as it does not pose neurological risks or elevate blood sugar levels (Goyal et al., 2010).

The clinical trials on hypertensive patients showed that stevioside lowered blood pressure (Sharma et al., 2009) and was not broken down into steviol by any of the digestive enzymes in the gastrointestinal tract (GIT) of humans or animals but was metabolized into steviol in the cecum by gut bacteria (Brahmachari et al., 2011). Following oral administration, stevioside, its aglycone steviol, and steviol metabolites were investigated in blood, feces, and urine samples collected after 3 days. Analysis revealed a minimal uptake of stevioside by the gastrointestinal tract, with detectable levels falling below the UV detectors limit to sensitivity. Notably, stomach enzymes did not exert a degradation effect on stevioside (Koyama et al., 2003). Within the colon, however, gut microbiota facilitated the complete degradation of any stevioside that reached this region, with steviol being the sole metabolite identified in the fecal sample (Wingard Jr et al., 1980). In blood plasma, stevioside, free steviol, and other unbound steviol metabolites were not detected. However, steviol glucuronide (SV glu) was present, reaching a peak concentration of 33 μg/mL (equivalent to 21.3 μg steviol/mL) (Geuns et al., 2007). Analysis of urine samples revealed the absence of stevioside and free steviol and confirmed the presence of SV glu. Urinary excretion levels reached up to 318 mg/24 h urine (equivalent to 205 mg steviol equivalents/24 h). No other steviol derivatives were observed in the urine (Geuns et al., 2006; Geuns et al., 2007). Finally, fecal analysis identified only free steviol, with no other steviol metabolites or conjugates detected, which suggests that steviol is primarily excreted as SV glu via the urinary system (Geuns et al., 2003; Geuns et al., 2004; Simonetti et al., 2004).

Stevioside also improved postprandial glucose hemostasis in T2D patients (Gougeon et al., 2004). Stevioside increased glucose uptake and oxidation in diabetic muscles by increasing GLUT-4 synthesis, similarly to metformin. Molecular docking analysis showed that stevioside binds more tightly to insulin receptors and proteins. Stevioside enhances glucose uptake in diabetic gastrocnemius muscles by stimulating specific proteins and receptors during the signaling pathway (Deenadayalan et al., 2021). The *in vivo* studies suggest that stevioside may be a promising herbal medicine for the treatment of T2D (Deenadayalan et al., 2021). The main aim of the current study, as compared to previous studies, was to investigate the high binding affinity of stevioside to various receptors and proteins associated with insulin without any side effects as a safe glucose control drug/approach for diabetic patients.

## 2 Methodology

### 2.1 Retrieval and preparation of proteins

The protein structures of GLUT-4, IR, Akt, and IRS-1 were obtained from the Protein Data Bank (PDB) using the PDB IDs: 7WSN, 4XLV, 1O6L, and 1K3A, respectively. These structures were visualized and analyzed using PyMOL (PyMOL molecular graphics system, version 2.0, Schrödinger, LLC) after removing water molecules. To prepare the target protein input file for docking simulation, polar hydrogen atoms were added to the PDB protein file, and ions, water molecules, subunits, and ligands were removed from the original structure file.

### 2.2 Validation of proteins

The 3D models of all proteins were acquired and assessed by the use of PROCHECK (Laskowski et al., 1993), which was obtainable from the SAVES server and also utilized by the ProSA server for validation (Wiederstein and Sippl, 2007). The Ramachandran (RC) plot, which compares the overall quality of the structure to a well-refined structure of comparable resolution, is provided by the PROCHECK server, which also assesses the stereochemical quality of the proteins (Carrascoza et al., 2014).

ProSA is a popular tool for examining possible mistakes in 3D protein models that come from theoretical models, protein engineering, or experimental elucidation. The software program provides an energy and Z-score map for the protein. Protein residues’ solvent exposure potential and distance-based methods are used to calculate the energy of the structure. The total model quality of the protein 3D structure is shown by the Z-score. In the ProSA energy plot, energy is displayed as a function of the amino acid sequence position and is utilized to determine the 3D model’s local quality. Negative values signify a stable and sound model, while positive models correspond to incorrect portions (Wiederstein and Sippl, 2007).

### 2.3 Selection of the ligand

The plant-based glycosidic compound (stevioside) is retrieved from PubChem (https://pubchem.ncbi.nlm.nih.gov) using a “similar structure” search (Kim et al., 2016). The physiochemical properties of the selected ligands were calculated, and their structures were loaded onto a workstation for docking studies to assess their anti-diabetic activity.

### 2.4 Protein active site evaluation

The Computed Atlas of Surface Topography of proteins (CASTp) was used to characterize the active sites of the GLUT-4, Akt, IR, and IRS-1 proteins (available online: http://sts.bioe.uic.edu/castp/calculation.html accessed on 20 March 2020) and analyzed using Chimera version 1.12. The binding pocket of each protein was characterized by volume, surface area, and the presence of cavities in a solvent (Tian et al., 2018).

### 2.5 Prediction of drug ADMET

ADMET (absorption, distribution, metabolism, excretion, and toxicity) is a crucial aspect of *in silico* drug design as it helps assess the pharmacokinetic and pharmacodynamic properties of potential drug candidates. A popular computational method for ADMET prediction is pkCSM (http://structure.bioc.cam.ac.uk/pkcsm), which provides a full range of features to assess drug toxicity, distribution, metabolism, and absorption (Abdullahi et al., 2022).

### 2.6 Molecular docking of proteins with the ligand

To determine the molecular basis of stevioside specificity for these targets, the 3D structures of the IR, GLUT-4, Akt, and IRS-1 proteins (target) in association with stevioside (ligand) were evaluated using computational ligand–target docking. PyRx and HDOCK were used for molecular docking, with the AutoDock Vina option selected based on the scoring function (Yan et al., 2020; Pawar and Rohane, 2021). Using grid-based atomic affinity potentials, the interaction energy between stevioside and IR, GLUT-4, Akt, and IRS-1 proteins was determined at every step of the docking procedure (Vilar et al., 2008).

## 3 Results

### 3.1 Retrieval and preparation of proteins

The RCSB PDB database was used to obtain the target proteins GLUT-4 (Supplementary Figure S1A), IR (Supplementary Figure S1B), Akt (Supplementary Figure S1C), and IRS-1 (Supplementary Figure S1D). X-ray crystallography was used to confirm the structures of all proteins. The A/B chains of each target protein were sequenced with chain lengths ranging from 328 to 520 amino acids. The samples were identified by their PDB IDs (7WSN, 4XLV, 1O6L, and 1K3A) and had the maximum (3,567) and the minimum number of atoms (2,548), respectively. The highest resolution was 3.31 and the lowest was 1.60 (Supplementary Table S1). In *in silico* protein, resolution refers to the clarity of atomic distances between amino acid residues when visualized in software. A higher resolution indicates a clearer molecular image (Qian et al., 2007). Since the target protein had a secondary structure with folds and consisted of one or more chains, its 3D structure was retrieved from a tertiary database (Rahman et al., 2020). The three protein structures were subjected to solvent sterilization, and the original ligands were modified using PyMol software to optimize the binding energy during molecular docking simulations (Baran et al., 2017; Supplementary Figure S1.

### 3.2 Protein validation

The Ramachandran plot of GLUT-4, Akt, IR, and IRS-1 proteins was obtained from the SAVES server (Supplementary Figure S2), while their plot statistics are presented in Table 1. The plot statistics of GLUT-4 indicated that 91.2% of amino acid residues were present in the most preferred region and 8.8% occurred in the extra allowed region according to the plot, while none of the amino acid residues was found in the regions that were liberally allowed or banned (Supplementary Figure S1A). The plot statistics of Akt protein indicated that 90.0% of the residues of amino acids were found in the most favored region, 7.6% were in the extra allowed region, 1.0% was in the generously allowed zone, and 1.4% was in the prohibited region (Supplementary Figure S2B). The plot data of IR protein revealed that 91.2% of amino acid residues were found in the most favorite region, 8.8% were in the extra permitted zone, and none were found in the regions that either liberally permitted or outlawed (Supplementary Figure S2C). Analysis of IRS-1 amino acid distribution using the Ramachandran plot revealed that 93.5% residues resided in the most favored regions while 6.2% were found in the additionally allowed regions. Only 0.4% of the residues occupied the generously allowed regions, and none were located in the disallowed regions (Supplementary Figure S2D). Hence, the created 3D models are good in terms of stereochemical quality.

**TABLE 1:** Ramachandran plot analysis of the residues in the GLUT-4, Akt, IR, and IRS-1 proteins occupies the most favored regions, indicating well-defined and stable secondary structures. Description, number of amino acids, and percentages for all proteins are given.

Description	No. of amino acids				Percentage			
	GLUT-4	Akt	IR	IRS-1	GLUT-4	Akt	IR	IRS-1
Residues in most favored regions	354	260	239	243	91.2%	90.0%	91.2%	93.5%
Residues in additional allowed regions	34	22	23	16	8.8%	7.6%	8.8%	6.2%
Residues in generously allowed regions	0	3	0	1	0.0%	1.0%	0.0%	0.4%
Residues in disallowed regions	0	4	0	0	0.0%	1.4%	0.0%	0.0%
Number of non-glycine and non-proline residues	388	289	262	260	100.0%	100.0%	100.0%	100.0%
Number of end-residues (excl. Gly and Pro)	2	5	4	4				
Number of glycine residues (shown as triangles)	49	21	21	22				
Number of proline residues	24	12	17	13				
Total number of residues	463	327	304	299				

The GLUT-4, Akt, IR, and IRS-1 proteins were submitted to the ProSA web server, and a Z-score of GLUT-4 protein was obtained at −6.98 (Supplementary Figure S3A), Z-score of Akt protein was obtained at −7.98 (Supplementary Figure S3B), the Z-score of IR protein was obtained at −8.74 (Supplementary Figure S3C), and the Z-score of IRS-1 protein obtained at −8.46 (Supplementary Figure S3D). The plot of the Z-scores of all protein chains in PDB as calculated via NMR spectroscopy (dark blue region) and X-ray crystallography (light blue region) is presented in Supplementary Figure S3. The energy profiles of GLUT-4 (Supplementary Figure S4A), Akt (Supplementary Figure S4B, IR (Supplementary Figure S4C), and IRS-1 (Supplementary Figure S4D) proteins obtained using the ProSA web server are shown in Supplementary Figure S4. The ProSA energy map derived for each model demonstrates that the maximum residues were located within the negative energy zone, indicating that each model is stable.

### 3.3 Ligand selection

**FIGURE 1:**
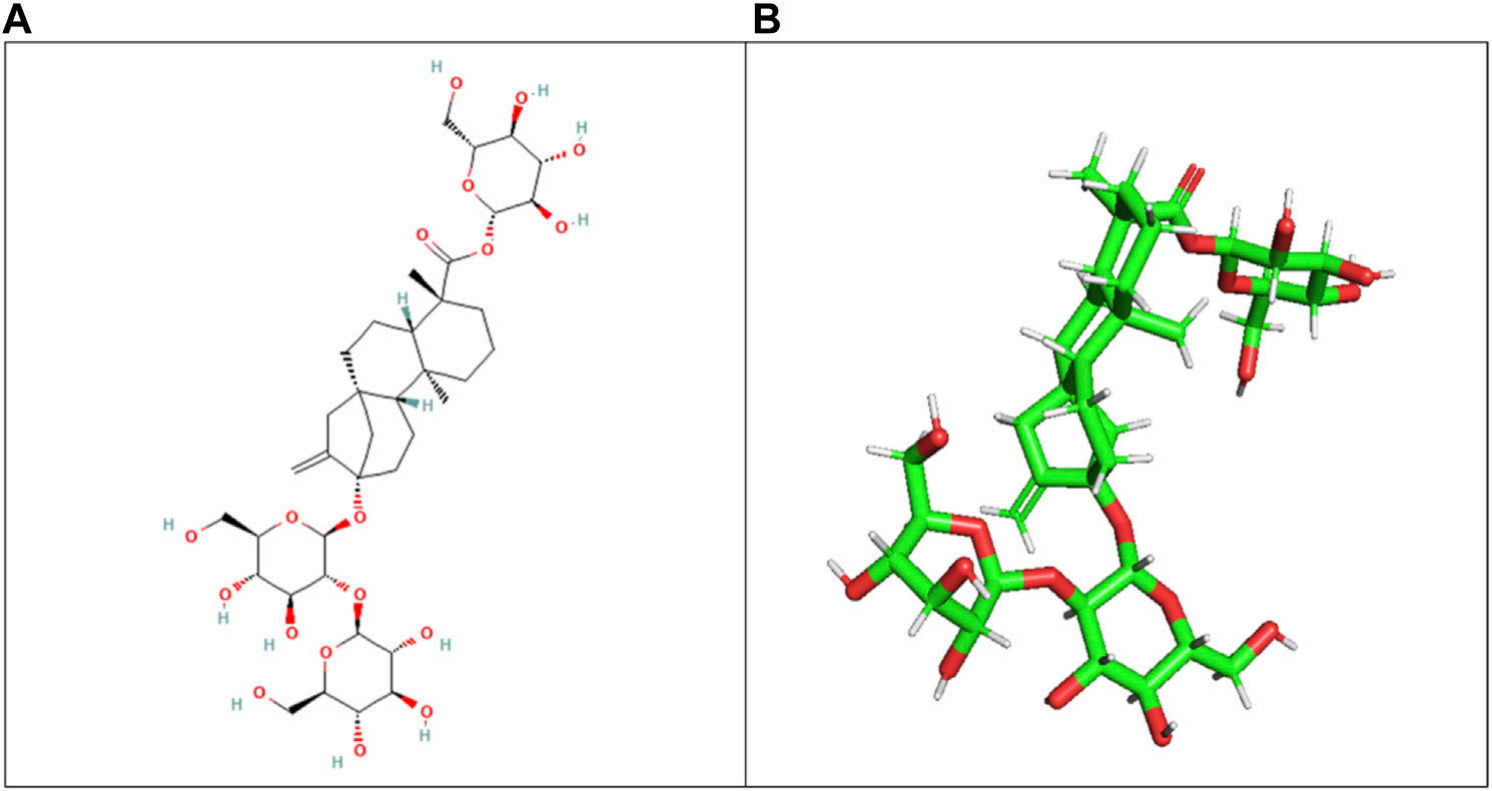
**(A)** 2D chemical structure of stevioside, a natural sweetener derived from the stevia plant, and **(B)** 3D visualization of stevioside, revealing its intricate molecular architecture.

The ligand (stevioside) obtained from PubChem consisted of PubChem CID 442089. The molecular weight of stevioside was 804.9 g/mol, the molecular formula of stevioside was C_38_H_60_O_18_, and the 2D and 3D structures of stevioside are shown in Figure 1.

### 3.4 Protein active site evaluation

The CASTp server was used to identify the amino acid residues that lined the binding site positions of GLUT-4, IR, Akt, and IRS-1 proteins. Additionally, three major binding pockets for each protein were predicted using CASTp. These pockets were subsequently analyzed using Chimera 1.12, respectively. Furthermore, binding pockets with a small surface area and volume were excluded from consideration as potential ligand-binding sites for virtual screening.

According to the CASTp server predictions, the GLUT-4 active site positions residues within pocket 1, the largest and most surface-exposed pocket on the GLUT-4 protein. This pocket was also lined with several residues that were known to be crucial for glucose transport, including Thr21, Leu24, Phe38, Ile42, Phe88, and Ser95. The GLUT-4 pocket 1 comprises 104 amino acid residues, including Thr21, Leu24, Ser35, Phe38, Ile42, Phe88, Ser95, Ser96, Phe97, Ile99, Gly100, Ile101, Ser103, Gln104, Arg108, Gly150, Thr152, Ser153, Gly154, Val156, Pro157, Met158, Val160, Gly161, Glul62, Ile163, Ala164, Pro165, Thr166, His167, Leu168, Arg169, Gly170, Leul72, Gly173, Thr174, Asn176, Gln177, Ile180, Val181, Ile184, Pro227, Arg228, Ile233, Leu244, Leu247, Thr248, Trp250, Val253, Val256, Glu259, Leu260 Asp262, Glu263, Lys266, Leu267, Thr337, Ser340, Val341, Val344, Glu345, Arg349, Phe395, Glu396, Gly400, Pro401, Trp404, Phe405, Asn427, Trp428, Asn431, Thr471, Thr471, Arg472, Gly473, Arg474, Thr475, Phe476, Asp477, and Ile479. Using CASTp software with default parameters, 50 active site positions were identified and analyzed within the GLUT-4 protein structure. All pockets were described to determine the residues surrounding them within a probe radius of 1.4 Ǻ. Of these, the biggest active site has a zone of 2158.359 Ǻ^2^ and a volume of 2765.094 Ǻ³ during analysis. The position of the largest active site in the protein, which ranges from amino acids 21 to 479, is shown in Supplementary Figure S5 and is highlighted in green.

During the study, pockets 2 and 3 were accurately predicted by CASTp as potential binding sites, but their likelihood of being active sites was lower than that of pocket 1. Pocket 2 was smaller and less exposed to the surface of the protein than pocket 1. It consists of 17 amino acid residues, namely, Gln49, Pro74, Thr78, Tyr309, Ser312, Ile313, Thr316, Leu371, Phe438, Gln439, Tyr440, Ala442, Glu443, Gly446, Pro447, Val449, and Phe450. The predicted active site of pocket 2 has a surface area of 152.196 Å^2^ and a volume of 73.774 Ǻ^3^.

**FIGURE 2:**
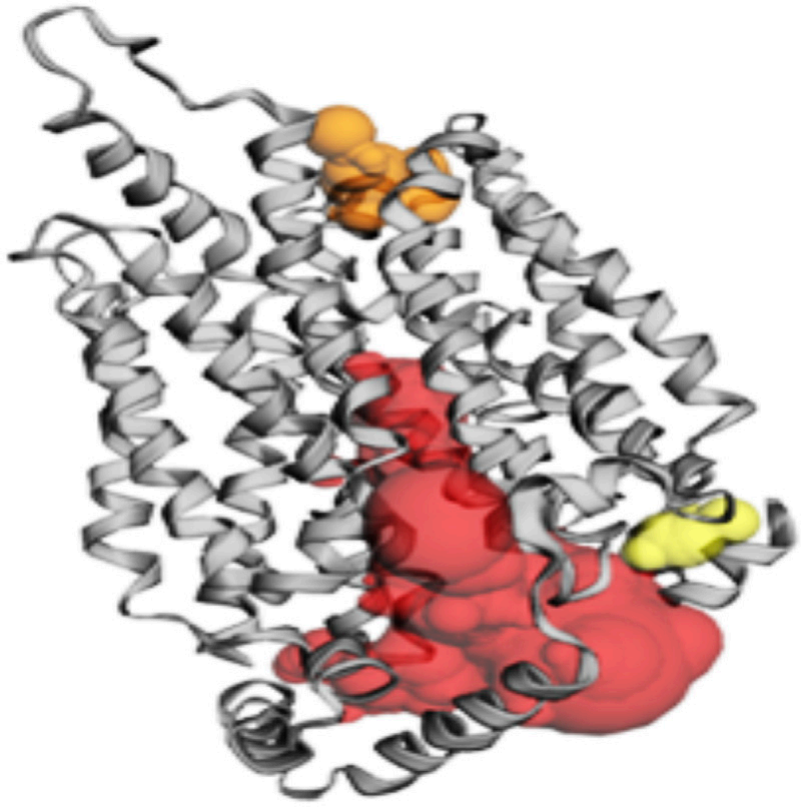
The gray color shows GLUT-4 protein; the red color shows pocket 1 with is the largest active site with 80 amino acid residues, the orange color represents pocket 2, and the yellow color indicates pocket 3 of the GLUT-4 protein.

Among the pockets on the GLUT-4 protein, pocket 3 was the smallest and least exposed to the surface. The residues that consistently form pocket 3 were Thr283, His284, Pro287, Leu410, Phe411, Ser412, Pro415, Phe476, Ile479, Ser480, and Phe483. Pocket 3 has an active site with an area of 83.119 Å^2^ and a volume of 32.407 Ǻ^3^. The three pockets predicted by the CASTp tool for the GLUT-4 protein are shown in Figure 2 and Table 2. Pocket 1 is represented in red, pocket 2 in orange, and pocket 3 in yellow.

**TABLE 2:** Active site of GLUT-4 top three pockets, their binding site, chain, amino acid residues, surface area, and volume.

Binding site	Chain	Amino acid residue	Surface area (SA)/Å^2^	Volume/Å^3^
Pocket 1	A	Thr21, Leu24, Ser35, Phe38, Ile42, Phe88, Ser95, Ser96, Phe97, Ile99, Gly100, Ile101, Ser103, Gln104, Arg108, Gly150, Thr152, Ser153, Gly154, Val156, Pro157, Met158, Val160, Gly161, Glu162, Ile163, Ala164, Pro165, Thr166, His167, Leu168, Arg169, Gly170, Leu172, Gly173, Thr174, Asn176, Gln177, Ile180, Val181, Ile184, Pro227, Arg228, Ile233, Leu244, Leu247, Thr248, Trp250, Val253, Val256, Glu259, Leu260 Asp262, Glu263, Lys266, Leu267, Glu270, Pro272, Leu273, Ser274, Leu275, Gln298, Gln299, Ile303, Asn304, Phe307, Tyr308, Asn333, Thr337, Ser340, Val341, Val344, Glu345, Arg349, Phe395, Glu396, Gly400, Pro401, Trp404, Phe405, Ala408, Glu409, Phe411, Ser412, Gln413, Gly414, Arg416, Pro417, Ala418, Met420, Ala421, Gly424, Asn427, Trp428, Asn431, Thr471, Thr471, Arg472, Gly473, Arg474, Thr475, Phe476, Asp477, and Ile479	2,158.359	2,765.094
Pocket 2		Gln49, Pro74, Thr78, Tyr309, Ser312, Ile313, Thr316, Leu371, Phe438, Gln439, Tyr440, Ala442, Glu443, Gly446, Pro447, Val449, and Phe450	152.196	73.774
Pocket 3		Thr283, His284, Pro287, Leu410, Phe411, Ser412, Pro415, Phe476, Ile479, Ser480, and Phe483	83.119	32.407

Based on the predictions made by the CASTp server for the Akt protein, pocket 1 of the Akt protein stands out as the largest and most surface-exposed pocket, and it has been identified as the active site. Akt protein pocket 1 includes 49 amino acid residues, namely, Leu158, Gly159, Lys160, Gly161, Thr162, Phe163, Gly164, Lys165, Val166, Ala179, Lys181, Leu183, Arg184, Val187, Ile188, Lys191, Glu193, His196, Thr197, Glu200, Leu204, Thr213, Phe227, Met229, Glu230, Tyr231, Ala232, Glu236, Phe239, Asp275, Lys277, Glu279, Asn280, Met282, Thr292, Asp293, Phe294, Gly295, Leu296, Tyr438, Phe439, Asp440, Phe443, Arg6, Thr7, Thr8, Ser9, Phe10, and Ala11. A total of 22 active sites were evaluated in the structure through CASTp software with ideal parameters. The biggest active site has an area of 579.259 Å^2^ and a volume of 355.567 Ǻ^3^. All pockets were characterized to determine their residues around the probe radius of 1.4 Ǻ. The protein’s biggest active site is located between amino acids 158 and 443, as shown in Supplementary Figure S6, in green color.

The CASTp server also predicted pockets 2 and 3 comparatively lesser than pocket 1 and also predicted that pocket 1 is more exposed than pocket 2 and consists of 16 amino acid residues such as Asp303, Gly304, Ala305, Thr306, Asp326, Tyr327, Gly328, Arg329, Ala330, Pro389, Lys390, Gly394, Gly395, Gly396, Pro397, and Asp399. The biggest active site has an area of 174.712 Å^2^ and a volume of 174.712 Ǻ^3^.

**FIGURE 3:**
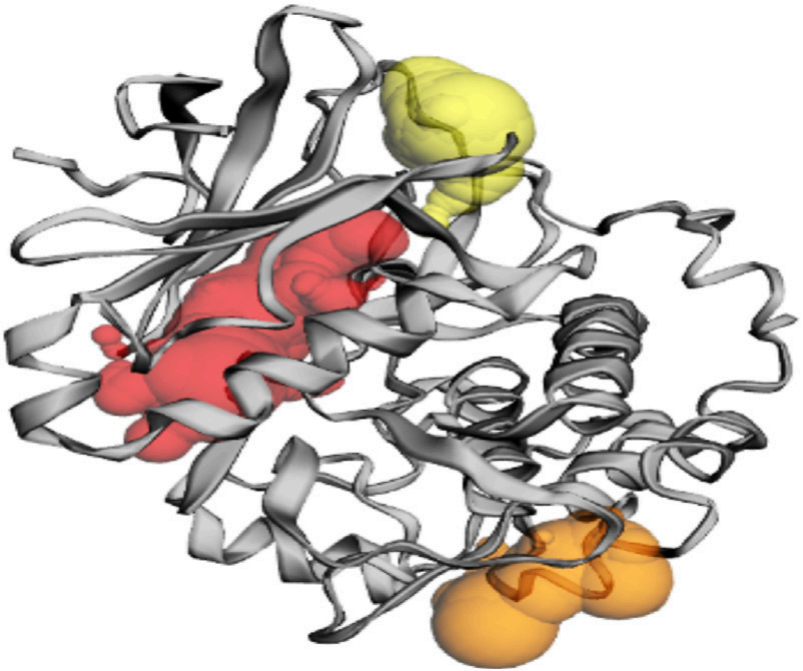
The gray color shows the Akt protein, the red color shows pocket 1 with the largest active site with 49 amino acid residues, the orange color represents pocket 2, and the yellow color indicates pocket 3 of the Akt protein.

Pocket 3 is the smallest and least surface-exposed pocket on the Akt protein; pocket 3 of the Akt protein consists of 13 residues such as Arg176, Tyr177, Tyr178, Thr213, Glu230, Tyr231, Ala232, Asn233, Asp284, Lys285, Lys290, Glu433, and Trp479. The biggest active site has an area of 121.624 Å^2^ and a volume of 81.980 Ǻ^3^. The three binding pockets were predicted for the Akt protein using the CASTp tool (Table 3; Figure 3). Furthermore, pocket 1 is highlighted in red, pocket 2 in orange, and pocket 3 in yellow.

**TABLE 3:** Active sites of Akt’s top three pockets, their binding site, chain, amino acid residues, surface area, and volume.

Binding site	Chain	Amino acid residue	Area (SA) Å^2^	Volume (SA) Å^3^
Pocket 1	A	Leu158, Gly159, Lys160, Gly161, Thr162, Phe163, Gly164, Lys165, Val166, Ala179, Lys181, Leu183, Arg184, Val187, Ile188, Lys191, Glu193, His196, Thr197, Glu200, Leu204, Thr213, Phe227, Met229, Glu230, Tyr231, Ala232, Glu236, Phe239, Asp275, Lys277, Glu279, Asn280, Met282, Thr292, Asp293, Phe294, Gly295, Leu296, Tyr438, Phe439, Asp440, and Phe443	579.259	355.567
Pocket 2	A	Asp303, Gly304, Ala305, Thr306, Asp326, Tyr327, Gly328, Arg329, Ala330, Pro389, Lys390, Gly394, Gly395, Gly396, Pro397, and Asp399	174.712	174.712
Pocket 3	A	Arg176, Tyr177, Tyr178, Thr213, Glu230, Tyr231, Ala232, Asn233, Asp284, Lys285, Lys290, Glu433, and Trp479	121.624	81.980

The CASTp server predicted the IR protein active sites (Table 4); pocket 1 was the largest and most surface-exposed pocket on the IR protein. The IR protein of pocket 1 consists of 53 amino acid residues, namely, Arg1000, Glu1001, Leu1002, Gly1003, Gln1004, Gly1005, Ser1006, Phe1007, Gly1008, Val1010, Glu1012, Ala1028, Lys1030, Thr1031, Val1032, Arg1039, Glu1040, Glu1043, Phe1044, Glu1047, Met1051, Val1060, Met1076, Glu1077, Leu1078, Met1079, Ala1080, His1081, Gly1082, Asp1083, Ser1086, Tyr1087, Ser1090, Asn1097, Pro1099, Arg1101, Arg1131, Asp1132, Arg1136, Asn1137, Met1139, Phe1144, Gly1149, Asp1150, Phe1151, Gly1152, Met1153, Arg1155, Lys1165, Gly1169, Leu1170, Leu1171, and Pro1172. Using the optimal settings provided by CASTp software, a total of 24 active sites within the structure were assessed. All the pockets were defined to determine their residues approximately at the probe radius of 1.4 Ǻ, and of them, the largest active site has a volume of 686.806 Ǻ^3^ and an area of 762.651 Å^2^, respectively. The biggest active site location in the protein was between 1000 and 1172 amino acids and highlighted in green (Supplementary Figure S7).

**TABLE 4:** Active sites of IR top three pockets, their binding site, chain, amino acid residues, surface area, and volume.

Binding site	Chain	Amino acid residue	Area (SA) Å^2^	Volume (SA) Å^3^
1	A	Arg1000, Glu1001, Leu1002, Gly1003, Gln1004, Gly1005, Ser1006, Phe1007, Gly1008, Val1010, Glu1012, Ala1028, Lys1030, Thr1031, Val1032, Arg1039, Glu1040, Glu1043, Phe1044, Glu1047, Met1051, Val1060, Met1076, Glu1077, Leu1078, Met1079, Ala1080, His1081, Gly1082, Asp1083, Ser1086, Tyr1087, Ser1090, Asn1097, Pro1099, Arg1101, Arg1131, Asp1132, Arg1136, Asn1137, Met1139, Phe1144, Gly1149, Asp1150, Phe1151, Gly1152, Met1153, Arg1155, Lys1165, Gly1169, Leu1170, Leu1171, and Pro1172	762.651	686.806
2	A	Arg1131, Lys1165, Gly1167, Lys1168, Gly1169, Leu1170, Leu1171, Pro1172, Val1173, Met1176, Ser1180, Leu1181, Gly1184, Phe1186, Asn1215, Glu1216, and Leu1219	215.893	254.117
3	A	Ile1019, Glu1022, Thr1025, Arg1026, Cys1056, His1057, Val1059, Arg1061, Leu1063, Glu1077, Leu1078, Met1079, Ala1080, Ala1141, His1142, Asp1143, Thr1145, and Lys1147	193.749	198.010

The prediction of the CASTp server proved that pocket 2 and 3 lesser extent than pocket 1. However, it has been observed that pocket 2 surface exposure was comparatively lower than pocket 1 and contains 17 amino acid residues such as Arg1131, Lys1165, Gly1167, Lys1168, Gly1169, Leu1170, Leu1171, Pro1172, Val1173, Met1176, Ser1180, Leu1181, Gly1184, Phe1186, Asn1215, Glu1216, and Leu1219. The biggest active site has an area of 215.893 Å^2^ and a volume of 254.117 Ǻ^3^.

**FIGURE 4:**
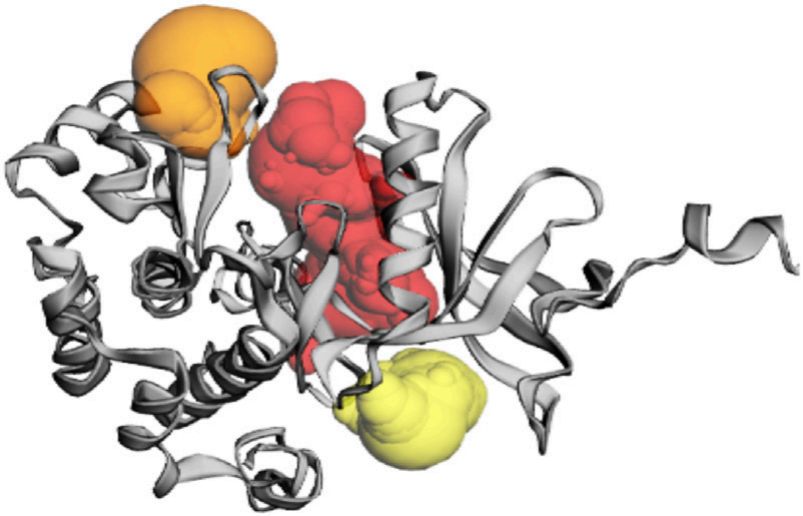
The gray color shows the IR protein, the red color shows pocket 1 with the largest active site with 53 amino acid residues, the orange color represents pocket 2, and the yellow color indicates pocket 3 of the IR protein.

Pocket 3 is the least surface-exposed pocket on the IR protein; pocket 3 of the IR protein consists of 18 residues, namely, Ile1019, Glu1022, Thr1025, Arg1026, Cys1056, His1057, Val1059, Arg1061, Leu1063, Glu1077, Leu1078, Met1079, Ala1080, Ala1141, His1142, Asp1143, Thr1145, and Lys1147. The biggest active site has an area of 193.749 Å^2^ and a volume of 198.010 Ǻ^3^. All three pockets were predicted by the CASTp tool of the IR protein (Figure 4); the red color represented pocket 1, the orange color represented pocket 2, and the yellow color represented pocket 3.

The CASTp server predicts the IRS-1 protein active sites (Table 5); pocket 1 was the largest and most surface-exposed pocket on the IRS-1 protein. The IRS-1 protein of pocket 1 consists of 59 amino acid residues, namely, Arg973, Leu975, Gly976, Gln977, Gly978, Ser979, Phe980, Gly981, Met982, Val983, Glu985, Arg999, Ala1001, Lys1003, Thr1004, Glu1013, Glu1016, Phe1017, Asn1019, Glu1020, Ala1021, Val1023, Met1024, Val1033, Leu1035, Val1047, Met1049, Glu1050, Leu1051, Met1052, Thr1053, Gly1055, Asp1056, Val1102, His1103, Arg1104, Asp1105, Arg1109, Asn1110, Cys1111, Met1112, Gly1122, Asp1123, Phe1124, Gly1125, Met1126, Thr1127, Arg1128, Ile1130, Thr1133, Asp1134, Arg1137, Lys1138, Gly1142, Leu1143, Leu1144, Val1158, Phe1159, and Thr1160. Using the optimal settings provided by CASTp software, a total of 35 active sites within the structure were assessed. All the pockets were defined to determine their residues approximately at the probe radius of 1.4 Ǻ, and of them, the largest active site has a volume of 1073.500 Ǻ^3^ and an area of 1047.925 Å^2^, respectively. The biggest active site location in the protein was between 973 and 1160 amino acids and highlighted in green (Supplementary Figure S8).

**TABLE 5:** Active sites of IRS-1 top three pockets, their binding site, chain, amino acid residues, surface area, and volume.

Binding site	Chain	Amino acid residue	Area (SA) Å^2^	Volume (SA) Å^3^
**1**	**A**	ARG973, LEU975, GLY976, GLN977, GLY978, SER979, PHE980, GLY981, MET982, VAL983, GLU985, ARG999, ALA1001, LYS1003, THR1004, GLU1013, GLU1016, PHE1017, ASN1019, GLU1020, ALA1021, VAL1023, MET1024, VAL1033, LEU1035, VAL1047, MET1049, GLU1050, LEU1051, MET1052, THR1053, GLY1055, ASP1056, VAL1102, HIS1103, ARG1104, ASP1105, ARG1109, ASN1110, CYS1111, MET1112, GLY1122, ASP1123, PHE1124, GLY1125, MET1126, THR1127, ARG1128, ILE1130, THR1133, ASP1134, ARG1137, LYS1138, GLY1142, LEU1143, LEU1144, VAL1158, PHE1159, and THR1160	1,073.500	1,047.925
**2**	A	HIS1030, HIS1031, LEU1057, TYR1060, LEU1061, LEU1064, PRO1077, LYS1081, GLN1084, MET1085, GLU1088, VAL1113, ASP1116, PHE1117, THR1118, VAL1119, GLU1241, GLY1243, PHE1244, and VAL1247	203.855	136.855
**3**	A	TRP962, VAL992, GLU995, THR998, ARG999, MET1024, LYS1025, PHE1027, ASN1028, CYS1029, HIS1030, VAL1032, VAL1033, ARG1034, LEU1035, LEU1036, GLU1050, LYS1120, and PHE1124	152.167	116.874

Although the CASTp server identifies pockets 2 and 3, these are predicted to be of lesser significance compared to pocket 1; pocket 2 consists of 20 amino acid residues, namely, His1030, His1031, Leu1057, Tyr1060, Leu1061, Leu1064, Pro1077, Lys1081, Gln1084, Met1085, Glu1088, Val1113, Asp1116, Phe1117, Thr1118, Val1119, Glu1241, Gly1243, Phe1244, and Val1247. The biggest active site has an area of 203.855 Å^2^ and a volume of 136.855 Ǻ^3^.

**FIGURE 5:**
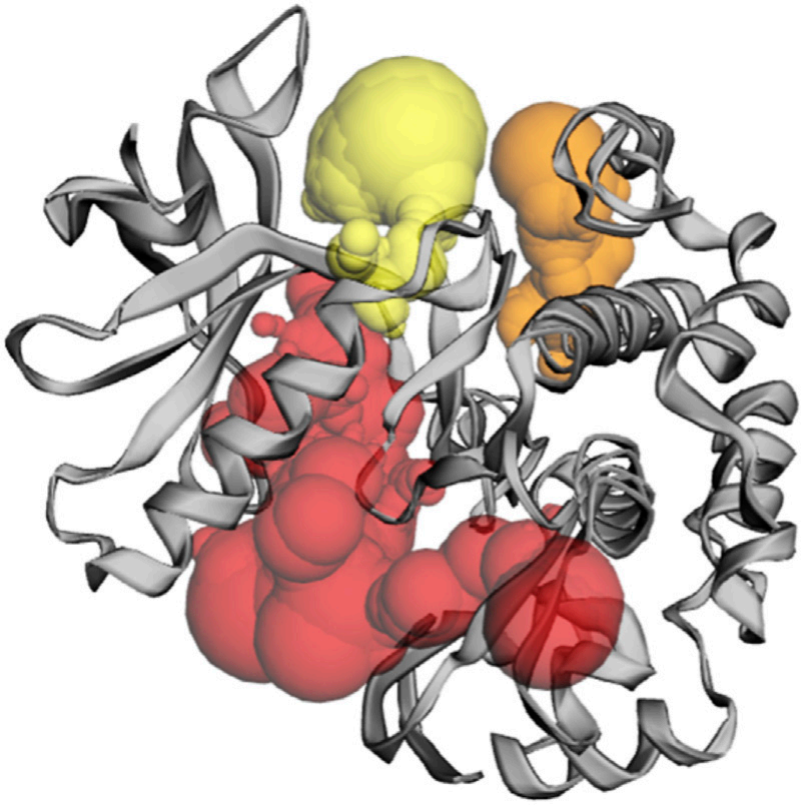
The gray color shows the IRS-1 protein, the red color shows pocket 1 with the largest active site with 59 amino acid residues, the orange color represents pocket 2, and the yellow color indicates pocket 3 of the IRS-1 protein.

Pocket 3 is the least surface-exposed pocket on the IRS-1 protein; the pocket 3 of the IRS-1 protein consists of 19 residues, namely, Trp962, Val992, Glu995, Thr998, Arg999, Met1024, Lys1025, Phe1027, Asn1028, Cys1029, His1030, Val1032, Val1033, Arg1034, Leu1035, Leu1036, Glu1050, Lys1120, and Phe1124. The biggest active site has an area of 152.167 Å^2^ and a volume of 116.874 Ǻ^3^. All three pockets were predicted by the CASTp tool of the IRS-1 protein (Figure 5); the red color represented pocket 1, the orange color represented pocket 2, and the yellow color represented pocket 3.

### 3.5 ADMET studies

The ADMET (absorption, distribution, metabolism, excretion, and toxicity) values for the compound under investigation have been computed using the web server pkCSM. The pharmacokinetic properties of the molecule should be taken into consideration as important variables throughout the virtual screening process rather than just focusing on enhancing the binding affinity and increasing specificity. Analysis has been done on the current status of theoretical models for predicting characteristics of drug absorption, such as Caco-2 permeability, intestinal absorption, and blood–brain partitioning. The importance of predictive and physical and chemical characteristic in evaluating passive drug absorption was emphasized.

The obtaining result of water solubility was found at −2.733, which indicates they are highly soluble in liquid medium, and the value of Caco-2 permeability was obtained at −0.366. The intestinal absorption (human) value of stevioside is 0, which means that it was not absorbed to blood through the intestine. The VDss (human) level range was −0.305 log L/kg, fraction unbound (human) was 0.379 Fu, BBB permeability score was −2.003 log BB, and CNS permeability value was −6.742 log PS. Drugs do not display inhibition or substrate against the CYP3A4 substrate, CYP2D6 substrate, CYP2C19 inhibitor, CYP1A2 inhibitor, CYP2C9 inhibitor, CYP3A4 inhibitor, and CYP2D6 inhibitor, and these enzymes were mostly present in the liver. Total clearance of stevioside was found at 0.746, and no renal OCT2 substrate was present, respectively (Supplementary Table S2).

### 3.6 Aquatic and non-aquatic toxicity

The study of medication toxicity to aquatic and non-aquatic species is characterized by its effects on these two groups of animals. The nature of drugs is chemical. Therefore, during production at a pharmaceutical or drug manufacturer’s factory, they may degrade and combine, which could be hazardous to the ecosystem. In addition, after being administered, they might have harmful or cancerous effects on human body, and this makes the current study very significant. The aforementioned medications do not cause hepatotoxicity, AMES toxicity, or skin sensitivity; nevertheless, ordinary metformin hydrochloride may cause these side effects. The highest level of oral rat acute toxicity was 2.591 mol/kg/day, the maximum level of oral rat chronic toxicity was 5.552 mg/kg/day, and the maximum tolerable dose was −0914 mg/kg/day. All of them suggest that these medications have improved pharmacokinetic and physiochemical characteristics (Supplementary Table S3).

### 3.7 Molecular docking studies of GLUT4 with stevioside

**FIGURE 6:**
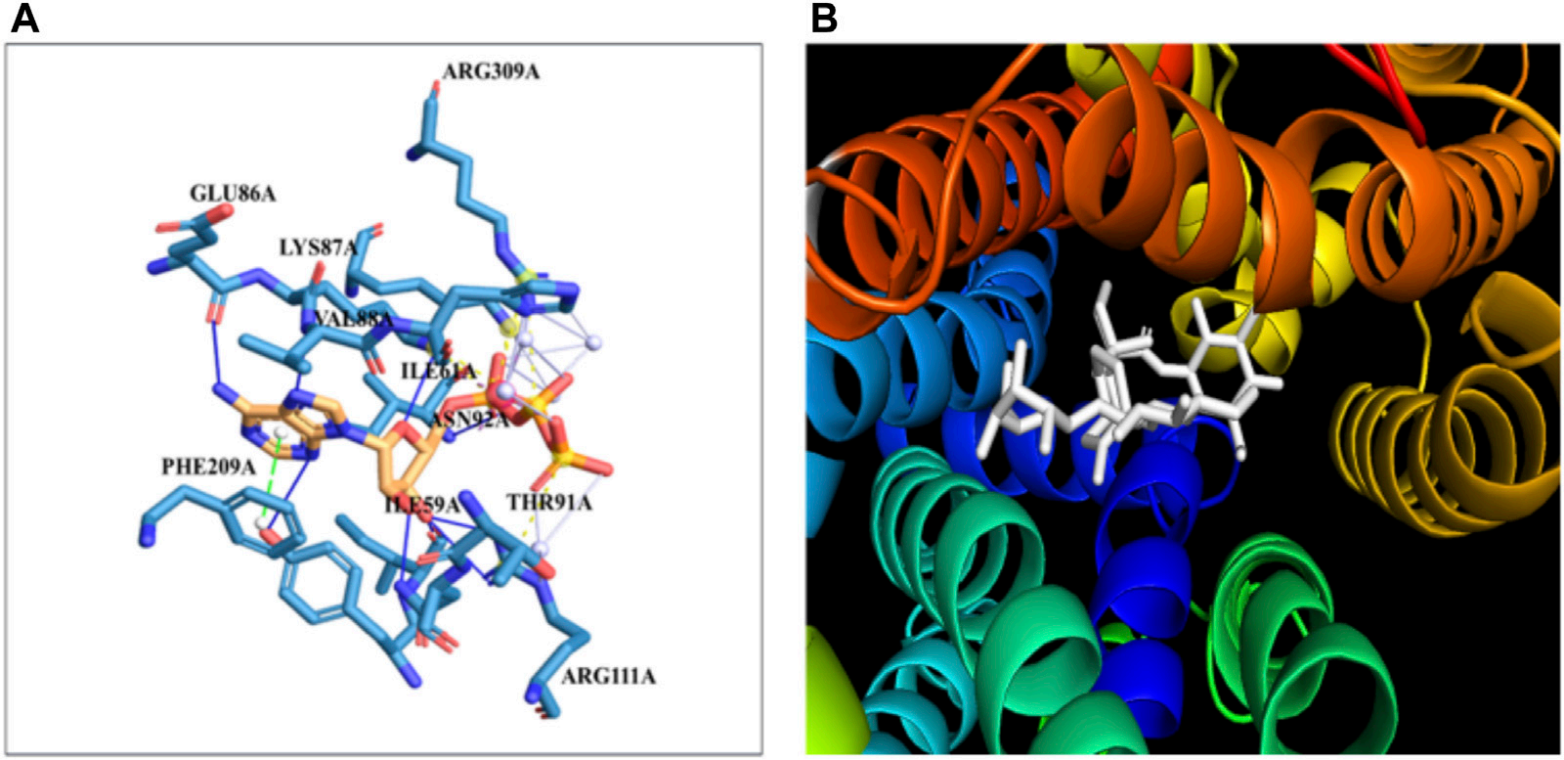
The binding of proteins and glycoside **(A)** GLUT-4 with the Stevioside, **(B)** the dot line shows salt bridges and blue line represents hydrogen bonds between Stevioside and GLUT-4 during binding.

The docking of the GLUT-4 protein with stevioside is predicted by the HDOCK server with a docking score of −327.28, with a confidence score of 0.9720 and ligand RMSD of 175.53 (Å) (Figure 6B). Here, the GLUT-4 docked position with stevioside reliably shows the stevioside binding position to the GLUT-4 protein (Figure 6A). Stevioside, in combination with GLUT-4, revealed a greater binding energy of −9.8 kcal/mol (Table 6) using PyRx software, with four salt bridges, and developed eight hydrogen bond interactions with amino acids, namely, TYR-58, ILE-59, ILE-61, GLU-86, LYS-87, VAL-88, ASN-92, and ARG-111, possessing hydrogen bond distances of 3.26 Å, 1.91 Å, 1.87 Å, 2.03 Å, 2.81 Å, 2.09 Å, 1.92 Å, and 1.88 Å, respectively.

**TABLE 6:** Docking affinity score, number of salt bridges, number of hydrogen bonds, and the distances of hydrogen bonds of stevioside with GLUT-4, Akt, IR, and IRS-1 proteins.

Protein name	Docking affinity	Salt bridge	Hydrogen bond	Distance
GLUT4	−9.8	LYS-87 ARG-111 LYS-307 ARG-309	TYR-58 ILE-59 ILE-61 GLU-86 LYS-87 VAL-88 ASN-92 ARG-111	3.26 1.91 1.87 2.03 2.81 2.09 1.92 1.88
Akt	−6.7	LYS-285	GLY-175 ARG-176 TYR-177 GLU-230 TRP-479	3.09 2.98 1.77 2.22 2.03
IR	−8	HIS-1057 ARG1061 LYS-1147	CYS-1056 HIS-1057 GLU-1077 GLN-1111 GLU-1115 HIS-1142 ASP-1143 LYS-1147 HIS-1268 SER-1270	2.49 2.33 2.02 3.18 3.30 2.77 1.98 3.20 2.77 2.50
IRS-1	−8.8	ARG-1245	GLU-1238 TYR-1250 TYR-1251 ASN-1255	3.22 1.91 1.52 3.19

### 3.8 Molecular docking studies of Akt with stevioside

**FIGURE 7:**
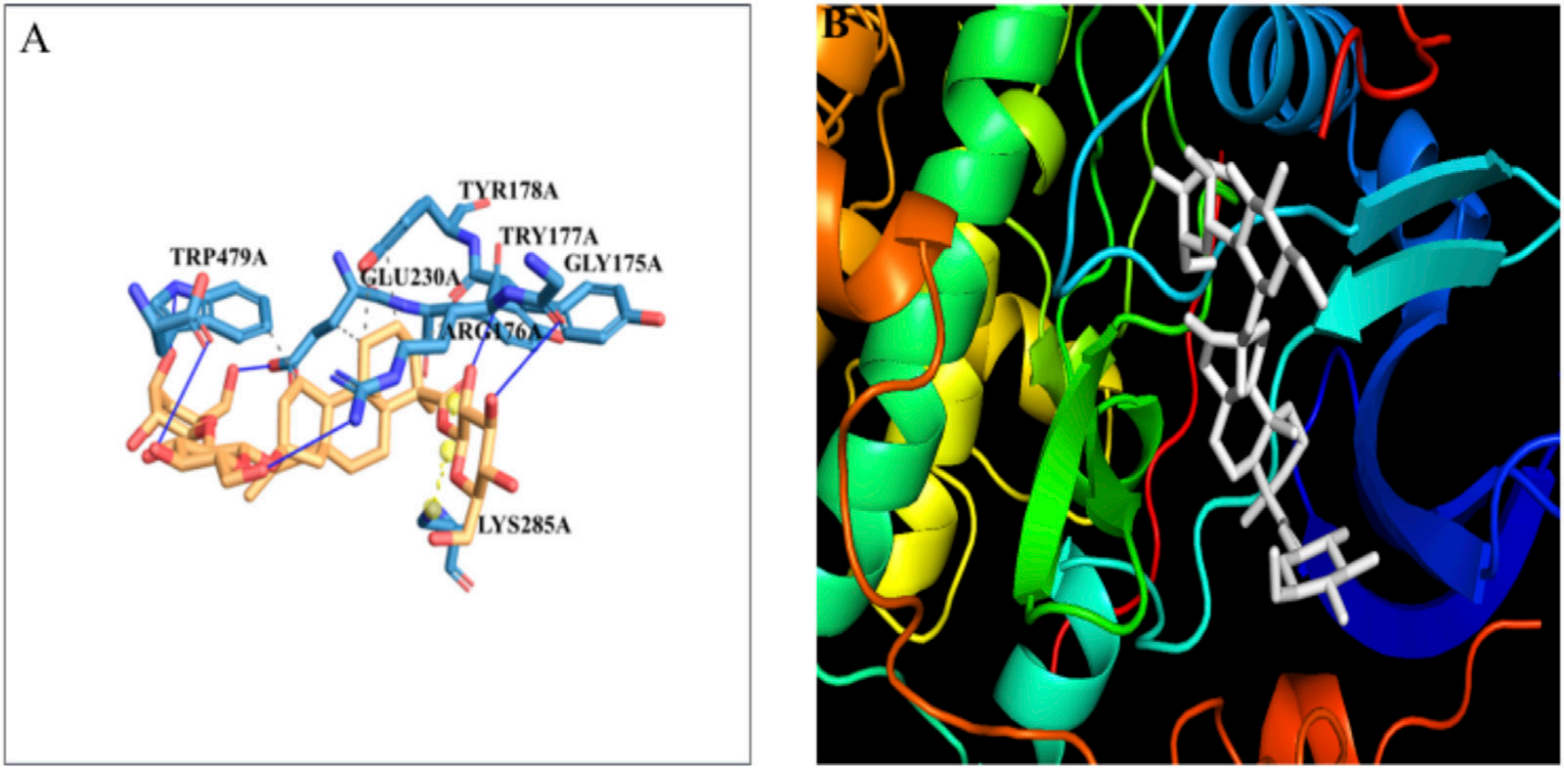
**(A)** the blue line represents hydrogen bonds and the orange dot line shows salt bridges during binding, **(B)** visualization of the binding interaction of Akt with Stevioside.

The docking of the Akt protein with stevioside by the HDOCK server with the docking score was recorded as −212.94, along with a confidence score of 0.7788 and ligand RMSD of 130.23 (Å) (Figure 7B). Furthermore, the Akt protein docked region with stevioside demonstrated the Akt–stevioside-binding location (Figure 7A). Stevioside with Akt using PyRx software revealed higher binding energy at −6.7 kcal/mol, with one salt bridge, and formed five hydrogen bond interactions with amino acids (GLY-175, ARG-176, TYR-177, GLU-230, and TRP-479) with the hydrogen bond lengths of 3.09 Å, 2.98 Å, 1.77 Å, 2.22 Å, and 2.03 Å, respectively (Table 6).

### 3.9 Molecular docking studies of IR with stevioside

**FIGURE 8:**
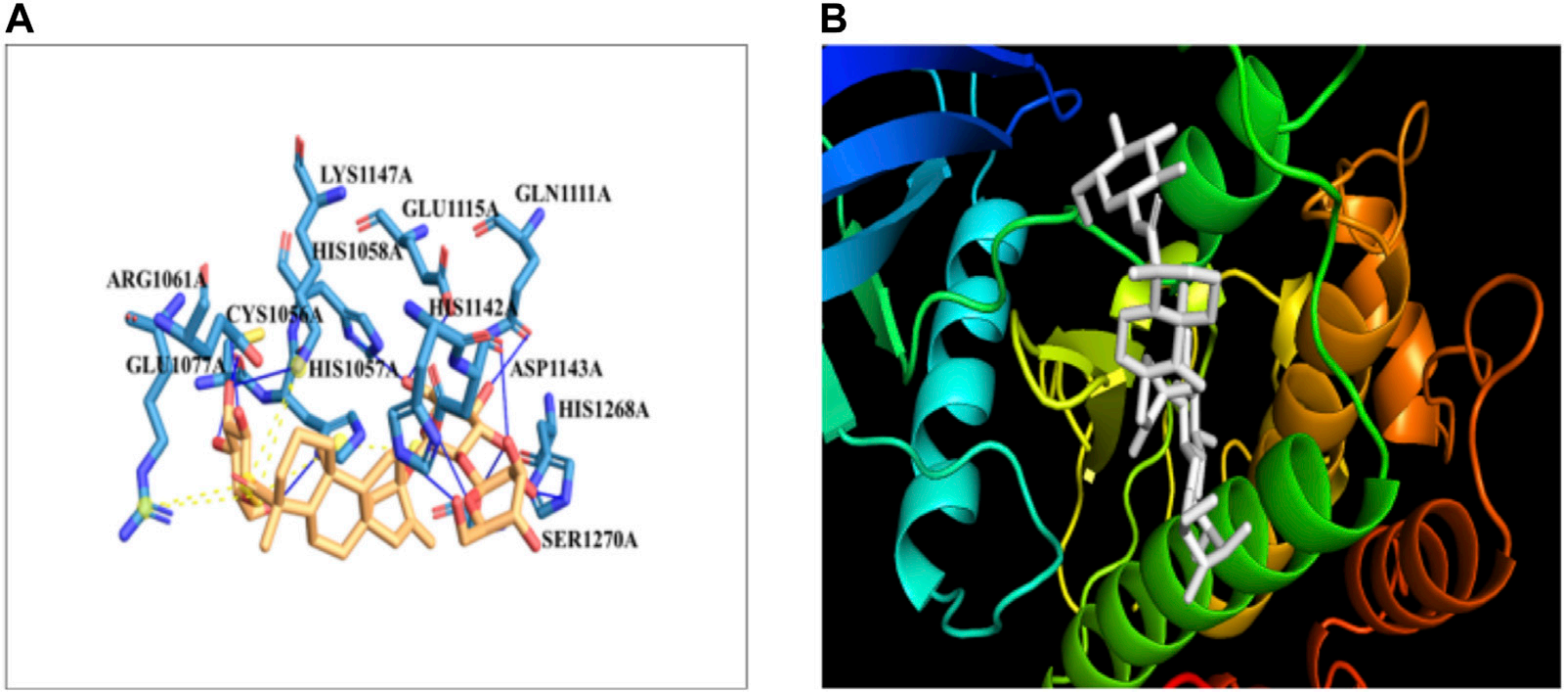
**(A)** Stevioside form 10 hydrogen bonds with IR protein, a blue line represents hydrogen bonds and an orange dot line shows salt bridges between IR and Stevioside, **(B)** the binding interaction of IR with Stevioside.

The docking of the IR protein with stevioside by the HDOCK server with the docking score was recorded as −244.64, along with a confidence score of 0.8691 and ligand RMSD of 74.83(Å) (Figure 8B). The IR protein docked site with stevioside accurately indicated the stevioside binding region to the IR protein (Figure 8A). Utilizing IR protein analysis using PyRx software, stevioside revealed a greater binding energy of −8 kcal/mol, with three salt bridges, and established 10 hydrogen bond connections with amino acids, namely, CYS-1056, HIS-1057, GLU-1077, GLN-1111, GLU-1115, HIS-1142, ASP-1143, LYS-1147, HIS-1268, and SER-1270 with hydrogen bond distances of 2.49 Å, 2.33 Å, 2.02 Å, 3.18 Å, 3.30 Å, 2.77 Å, 1.98 Å, 3.20 Å, 2.77 Å, and 2.50 Å correspondingly (Table 6).

#### 3.9.1 Molecular docking studies of IRS-1 with stevioside

**FIGURE 9:**
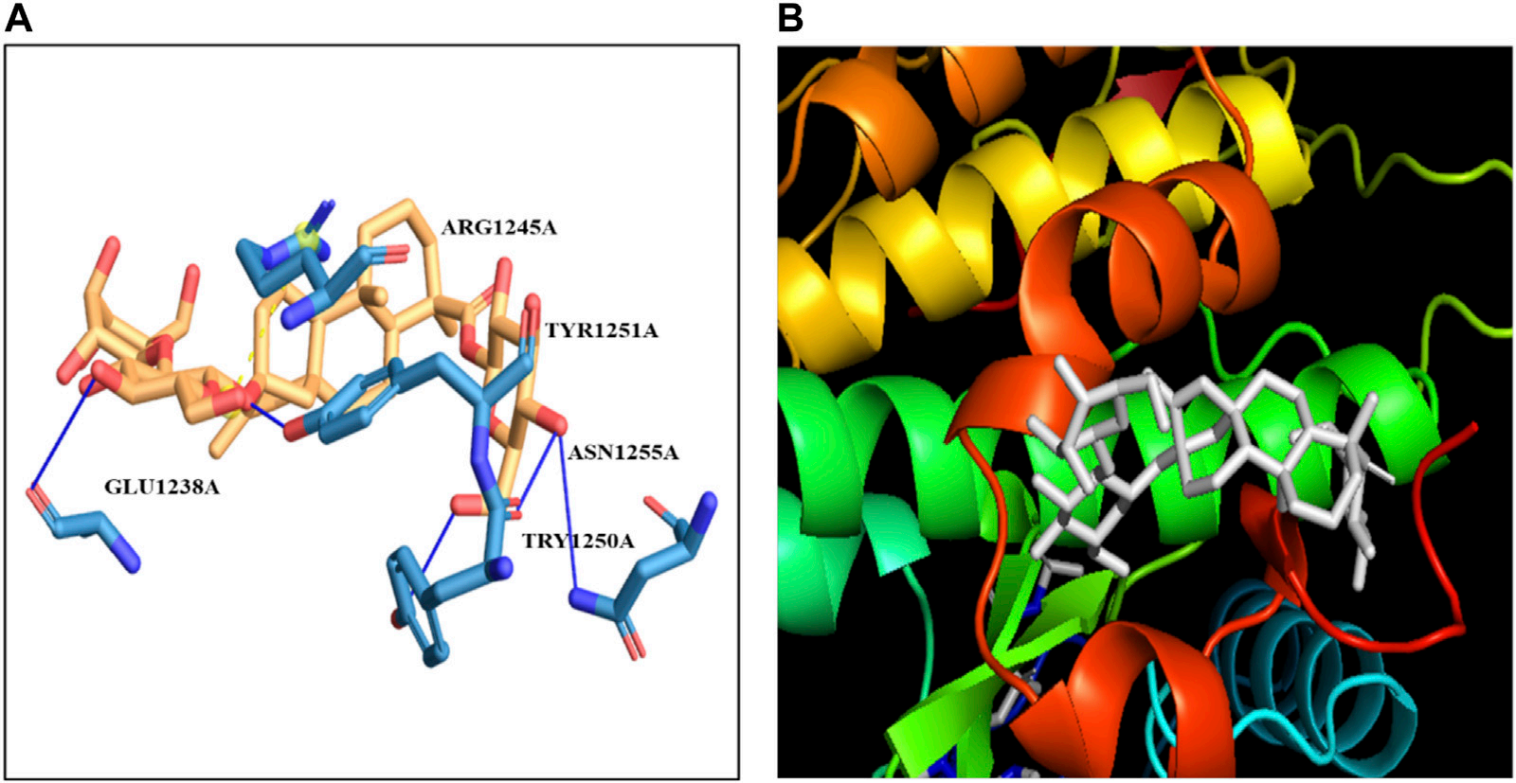
**(A)** the figure show one salt bridge and 4 hydrogen bonds where a blue line represents hydrogen bonds and an orange dot line shows salt bridges between IRS-1 and Stevioside. **(B)** the docking of Stevioside and IRS-1.

*In silico* docking analysis using the HDOCK server revealed that stevioside binds to the IRS-1 protein with a docking score of −201.97, a confidence score of 0.7387, and a ligand RMSD of 35.82 Å (Figure 9B). The docking conformation of stevioside on the IRS-1 protein surface (Figure 9A) accurately depicts its binding region.

Further analysis using PyRx software indicated a strong binding interaction between stevioside and the IRS-1 protein. Stevioside exhibited a favorable binding energy of −8.8 kcal/mol and formed several key interactions with the protein. Notably, it established one salt bridge (ARG-1245) and four hydrogen bonds with specific amino acid residues, namely, GLU-1238, TYR-1250, TYR-1251, and ASN-1255. The hydrogen bond distances were measured to be 3.22 Å, 1.91 Å, 1.52 Å, and 3.19 Å, respectively (Table 6).

## 4 Discussion

Diabetes Mellitus Association’s stark forecast highlights the impending diabetes crisis, with 578 million cases expected in 2030 and a staggering 51% increase to 700 million by 2045 (Saeedi et al., 2019). It is distinguished by absolute or relative deficits in insulin production (T1D) and/or (T2D), which are linked to persistent hyperglycemia and abnormalities in the metabolism of proteins, lipids, and carbohydrates (Khan et al., 2012).

The impact of insulin was mimicked by stevioside acting as a receptor ligand. Rat fibroblasts responded to SGs by taking up more glucose (Prata et al., 2017). SGs increased the activity of the glucose transporter in human leukemia cells HL-60 and human neuroblastoma cells SH-SY5Y, similar to that of insulin action. Additionally, PI3K and Akt phosphorylation were enhanced by SGs and insulin. Therefore, it was suggested that the modification of the PI3K/Akt pathway is connected to GLUT translocation. Stevioside has been shown to improve insulin-mediated glucose transport into skeletal muscle and insulin sensitivity in rats that are both insulin-sensitive and insulin-resistant. SGs restored normal lipid metabolism and prevented internal organ damage (Lailerd et al., 2004).

Sweeteners made from stevia extracts are widely used to sweeten a wide range of food and beverage products. They are available in powder, tablet, and liquid forms on the market (Ashwell, 2015). Steviol glycosides do exhibit exceptional chemical and physical stability, which permits their usage in acidic beverages, prepared meals such as biscuits and baked goods, dressings and sauces, frozen foods, processed fruits and vegetables, snacks, and cereals (Ciriminna et al., 2019). Some reports suggested that stevioside as a natural sweetener and sugar alternative will replace the synthetic sweeteners that is harmful and are carcinogenic (Ciriminna et al., 2019; Naz et al., 2024). As previously stated, more new commercial goods sweetened with steviol glycosides than with aspartame were introduced in 2017 (Ciriminna et al., 2019). Similar to glycosides, pectin is another source of carbohydrate with high commercial and market demand as natural sweetener, but it is also increase blood sugar level (Pagliaro et al., 2016). As per a well-known market research firm, the worldwide stevia market was valued at $338 million in 2015 and was projected to reach $554 million by 2024 (with a compound annual growth rate of 6.0%) (MVM, 2020).

A computational method called “molecular docking” aims to create a non-covalent binding between a tiny molecule (ligand) and a protein (receptor). The process of docking established the mechanism of interaction between the small ligand for the binding site and the target protein. The specific ligand’s affinity and potency with which the molecule interacts and binds to the target protein’s cavity are indicated by the binding energy. A compound that has a reduced binding energy is preferable as a possible therapeutic option. PyRx and HDOCK servers were used in diabetes molecular docking to determine the effect of stevioside against GLUT-4, Akt, IR, and IRS-1 proteins. Docking analysis of the stevioside compound showed the highest docking energy with GLUT-4, Akt, IR, and IRS-1 proteins and was the strongest molecule at the target protein site (Table 6). It has been demonstrated conclusively that the stevioside molecule is firmly attached to the chosen proteins, and all complexes appear to have more than five hydrogen bond interactions.

Using CASTp software with default parameters, 50 active site positions were identified and analyzed within the GLUT-4 protein structure, 22 active sites were evaluated in the Akt structure, 24 active sites within the structure of IR, and 35 active sites were within the structure of IRS-1. All pockets were described to determine the residues surrounding them within a probe radius of 1.4 Ǻ. The biggest active sites of GLUT-4, Akt, IR, and IRS-1 have a zone of 2,158.359 Ǻ^2^, 579.259 Ǻ^2^, 762.651 Ǻ^2^, and 1,073.500 Ǻ^2^ and a volume of 2,765.094 Ǻ³, 355.567 Ǻ³, 686.806 Ǻ³, and 1,047.925 Ǻ³, respectively.

Gastric juice and digestive enzymes are unable to break down stevioside, according to preclinical and clinical research (Hutapea et al., 1997; Koyama et al., 2003). Furthermore, because of its large molecular weight, oral stevioside does not appear to be absorbed at the upper small intestine level (Koyama et al., 2003). After taking 750 mg of stevioside per day, research including human volunteers revealed no detectable levels of stevioside, free steviol, or any other steviol metabolite in the blood. Still, the stool includes steviol (Geuns et al., 2007). The intestinal absorption (human) value of stevioside is 0, which means it does not absorb. The aforementioned medications do not cause hepatotoxicity, AMES toxicity, or skin sensitivity.

The *in silico* and *in vivo* studies suggested that stevioside may be a promising herbal medicine for the treatment of T2D (Deenadayalan et al., 2021). However, the current study focused on the binding affinity of stevioside with all the receptors and proteins involved in the insulin biosynthetic pathway through molecular docking and simulation and confirmed that stevioside is one of the potent natural drugs for diabetic patients that might be adopted by pharmaceutical industries for effective formulations.

No doubt, CADD also play an important role in drug discovery and drug designing and is comparatively cost effective than IA and ML tools (Vemula et al., 2023). CADD are rapid and advanced techniques that can be used in multiple fields, including biological sciences (Niazi and Mariam, 2024). It is considered one of the best tools for ligand–receptor interactions at various drug development stages (Wu et al., 2024).

Currently, sucrose is frequently substituted with the non-nutritive sweetener’s aspartame, acesulfame potassium, and saccharine to treat conditions including obesity, T2D, and hypertension. However, there is always concern about their negative effects, particularly in comparison to neurological impacts, carcinogenesis, increased hunger, and other issues. Thus, there is a growing need for new, secure, non-caloric, and non-cariogenic natural sweeteners. Naturally occurring stevioside may be utilized in conjunction with commercially accessible anti-diabetic medications as an adjuvant or alternative therapy for the treatment of diabetes.

## 5 Conclusion

The presence of sucrose in various dietary products (natural/synthetic) is one the major issues for public health, especially for diabetic patients. The resistant antibiotic is another problem in treating the infections of diabetic patients. The complete cure or treatment of diabetic patients is not possible; however, changing lifestyle, misuse of drugs, and proper medication can minimize the glucose level in the blood. Such escalating issues need alternative strategies to reduce the risk of diabetes in general public. Therefore, the current study declares that stevioside is one of the natural sweeteners that can be used by diabetic patients in various formulations rather than synthetic drugs to normalize their glucose level. With its impressive binding affinity, safety profile, and natural origin, stevioside holds significant promise for revolutionizing diabetes management. Its potential to mimic insulin’s action without adverse effects opens exciting avenues for future research and development. This study paves the way for incorporating stevioside into various dietary products, offering diabetics a safe and potentially effective sugar substitute. This study recommends that stevioside needs to be properly formulated by pharmaceutical industries to minimize the use of the synthetic drugs.

## Data Availability

The original contributions presented in the study are included in the article/Supplementary Material; further inquiries can be directed to the corresponding authors.
