# Supplementary figures and images for: Exploring stevioside binding affinity with various proteins and receptors actively involved in the signaling pathway and a future candidate for diabetic patients
Source: Front Pharmacol. 2024 Aug 7;15:1377916. doi: 10.3389/fphar.2024.1377916 (PMC11335537; doi:10.3389/fphar.2024.1377916)

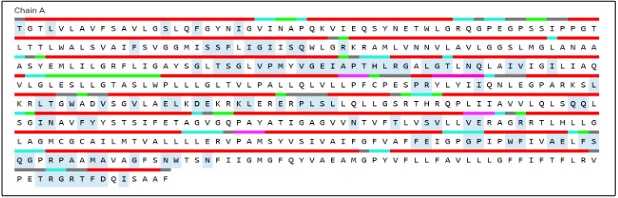

Supplement: Supplementary file 1 [file Image5.jpg]

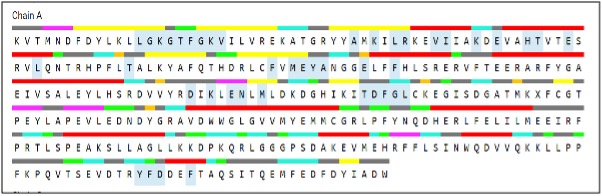

Supplement: Supplementary file 2 [file Image6.jpg]

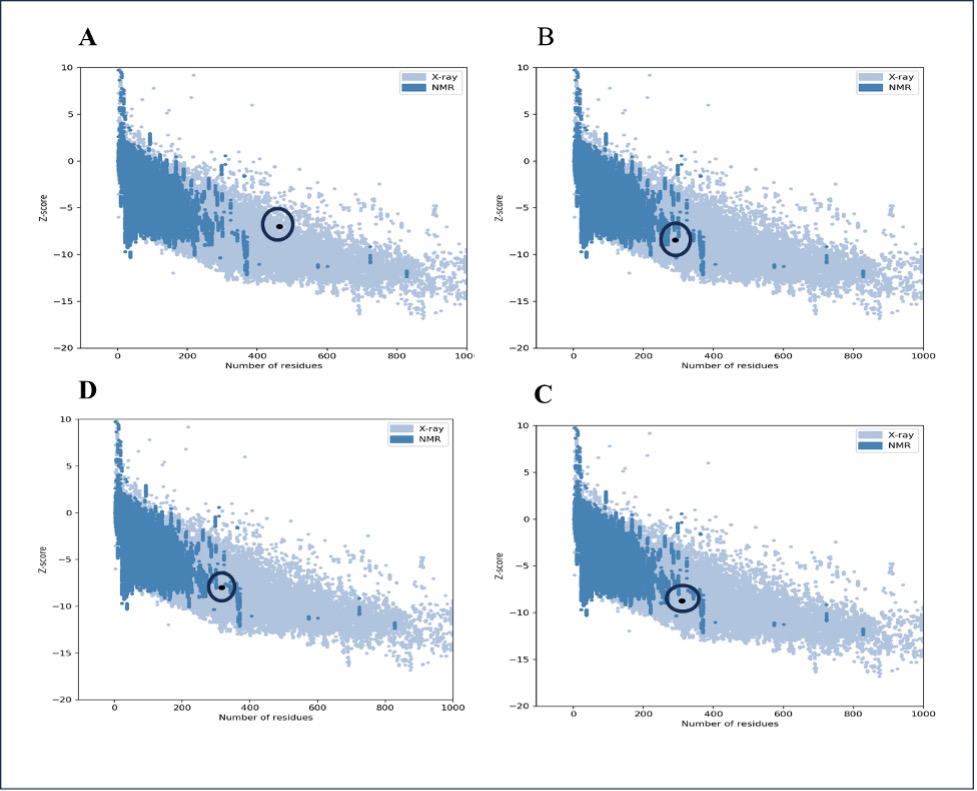

Supplement: Supplementary file 3 [file Image3.jpg]

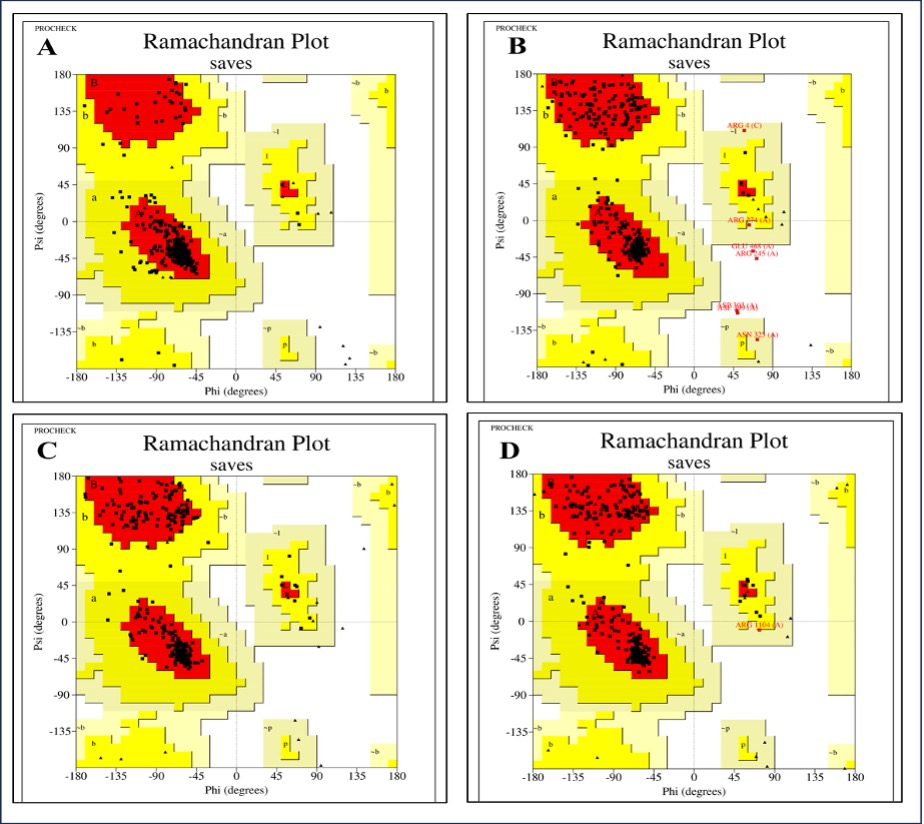

Supplement: Supplementary file 4 [file Image2.jpg]

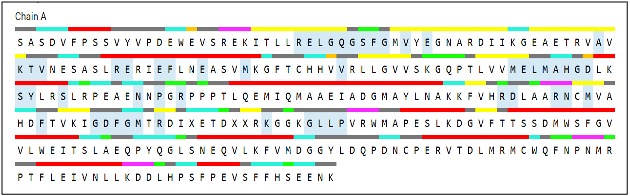

Supplement: Supplementary file 6 [file Image7.jpg]

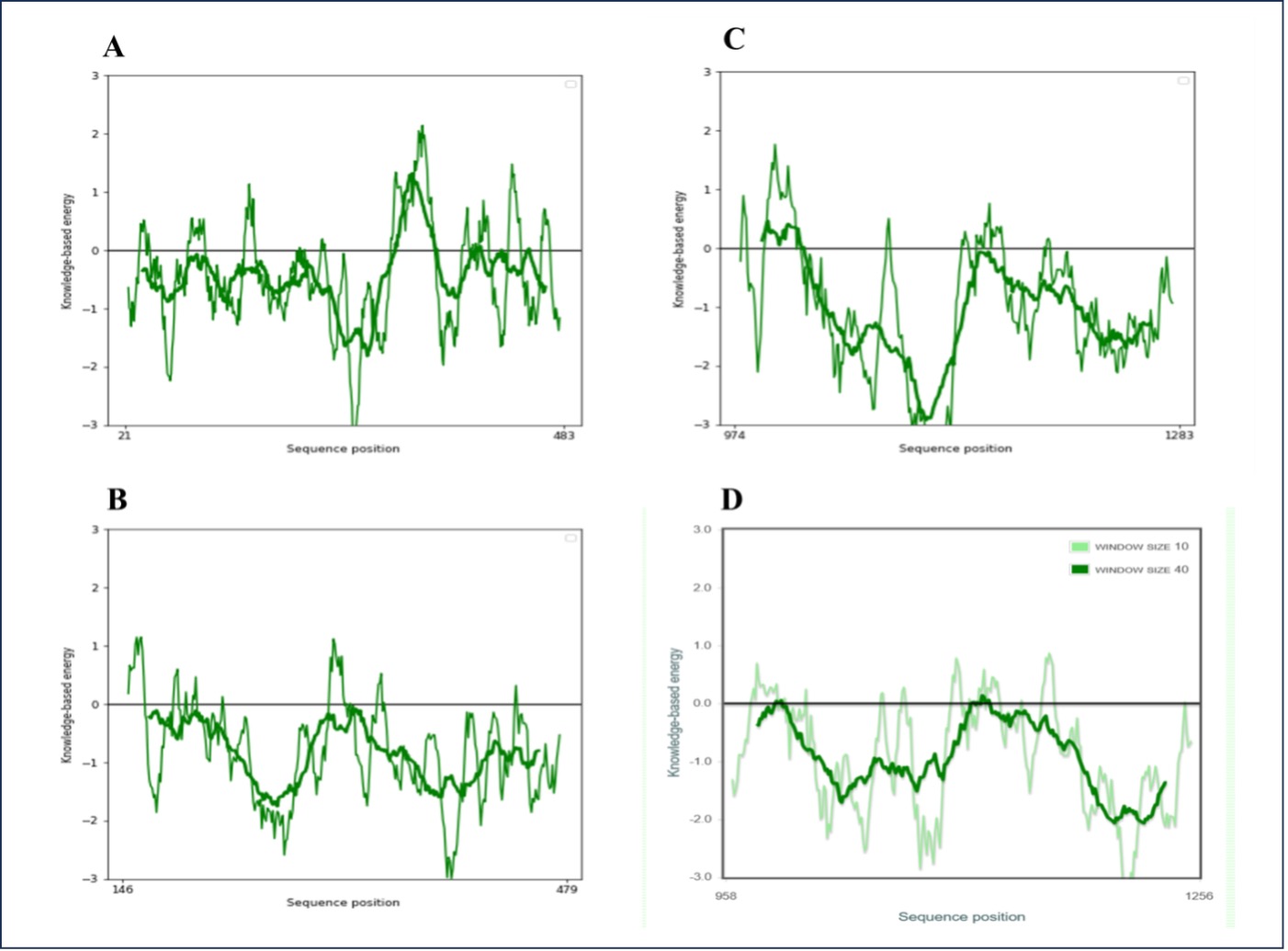

Supplement: Supplementary file 7 [file Image4.jpg]

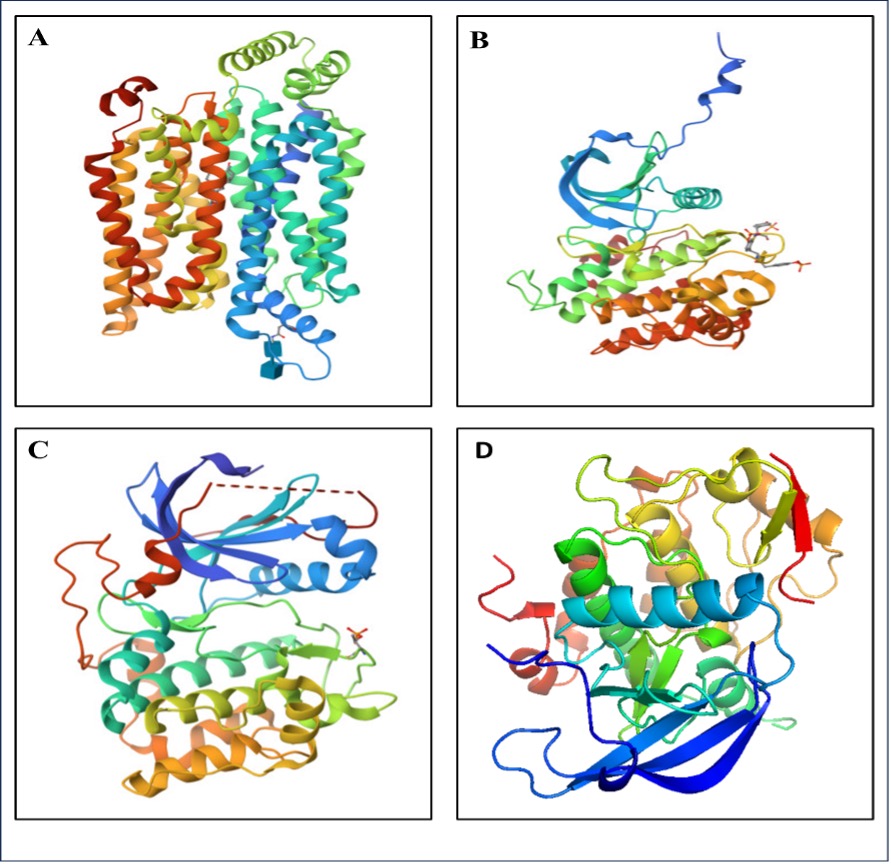

Supplement: Supplementary file 8 [file Image1.jpg]

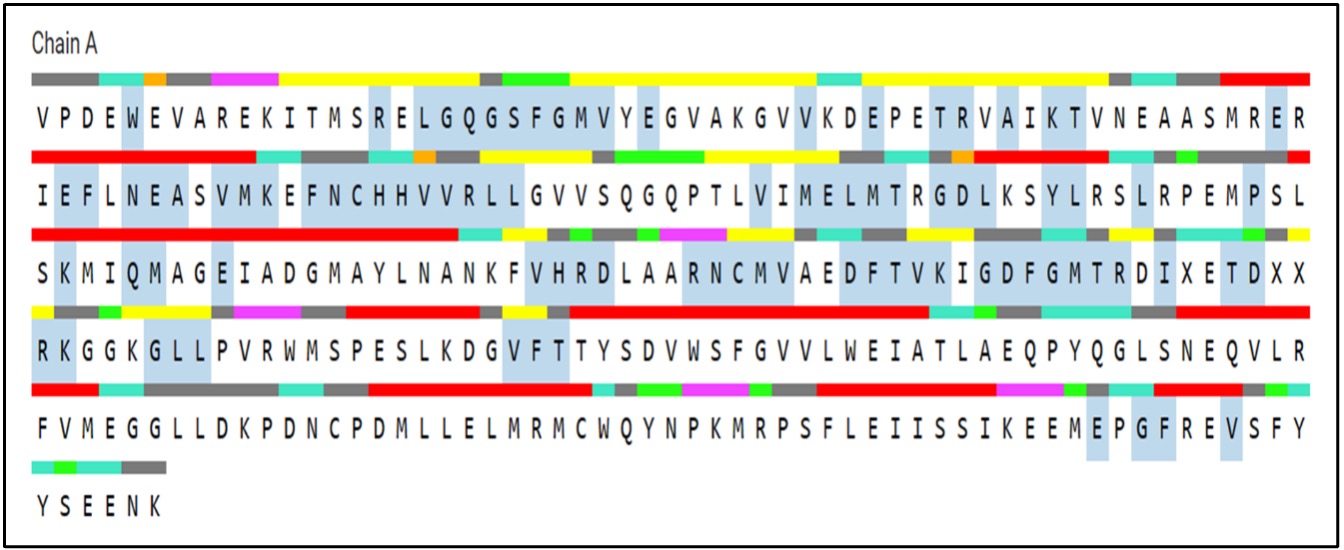

Supplement: Supplementary file 9 [file Image8.jpg]
