# Supplementary material for: Exploring stevioside binding affinity with various proteins and receptors actively involved in the signaling pathway and a future candidate for diabetic patients
Source: Front Pharmacol. 2024 Aug 7;15:1377916. doi: 10.3389/fphar.2024.1377916 (PMC11335537; doi:10.3389/fphar.2024.1377916)
Supplement: Supplementary file 5 [file Table1.docx]

**Supplementary file**

**Supplementary Table 1.** The results of target protein sample preparation from RCSB-PDB.

| **Name** | **PDB ID** | **Visualization** | **Resolution (Å)** | **Atom counts** | **Weight (kDa)** | **Chain** | **Sequence length (mer)** |
| --- | --- | --- | --- | --- | --- | --- | --- |
| GLUT4 | **7WSN** | X-ray | 3.31 | 3,567 | 57.01 | A | 520 |
| IR | **4XLV** | X-ray | 2.30 | 2,619 | 37.91 | A | 328 |
| Akt | **1O6L** | X-ray | 1.60 | 3,118 | 41.13 | A | 337 |
| IRS-1 | **1K3A** | X-ray | 2.10 | 2,548 | 36.51 | A | 299 |

**Supplementary Table 2.** Prediction of ADMET properties of Stevioside using pkCSM ADME.

| **Property** | **Model Name** | **Predicted Value** | **Unit** |
| --- | --- | --- | --- |
| Absorption | Water solubility | -2.733 | Numeric (log mol/L) |
| Absorption | Caco2 permeability | -0.366 | Numeric (log Papp in 10^-6^ cm/s) |
| Absorption | Intestinal absorption (human) | 0 | Numeric (% Absorbed) |
| Absorption | Skin permeability | -2.735 | Numeric (log Kp) |
| Absorption | P-glycoprotein substrate | Yes | Categorical (Yes/No) |
| Absorption | P-glycoprotein I inhibitor | No | Categorical (Yes/No) |
| Absorption | P-glycoprotein II inhibitor | No | Categorical (Yes/No) |
| Distribution | VDss (human) | -0.305 | Numeric (log L/kg) |
| Distribution | Fraction unbound (human) | 0.379 | Numeric (Fu) |
| Distribution | BBB permeability | -2.003 | Numeric (log BB) |
| Distribution | CNS permeability | -6.742 | Numeric (log PS) |
| Metabolism | CYP2D6 substrate | No | Categorical (Yes/No) |
| Metabolism | CYP3A4 substrate | No | Categorical (Yes/No) |
| Metabolism | CYP1A2 inhibitor | No | Categorical (Yes/No) |
| Metabolism | CYP2C19 inhibitor | No | Categorical (Yes/No) |
| Metabolism | CYP2C9 inhibitor | No | Categorical (Yes/No) |
| Metabolism | CYP2D6 inhibitor | No | Categorical (Yes/No) |
| Metabolism | CYP3A4 inhibitor | No | Categorical (Yes/No) |
| Excretion | Total Clearance | 0.746 | Numeric (log ml/min/kg) |
| Excretion | Renal OCT2 substrate | No | Categorical (Yes/No) |

**Supplementary Table 3.** Predicted toxicity profile of the Stevioside using pkCSM server.

| **Property** | **Model Name** | **Predicted value** | **Unit** |
| --- | --- | --- | --- |
| Toxicity | AMES toxicity | No | Categorical (Yes/No) |
| Toxicity | Max. tolerate dose (human) | -0.914 | Numeric (log mg/kg/day) |
| Toxicity | Oral Rat Acute Toxicity (LD50) | 2.591 | Numeric (mol/kg) |
| Toxicity | Oral Chronic Toxicity (LOAEL) | 5.552 | Numeric (log mg/kg/kg bw/day) |
| Toxicity | Hepatotoxicity | No | Categorical (Yes/No) |
| Toxicity | Skin Sensitization | No | Categorical (Yes/No) |
| Toxicity | T. Pyriformis toxicity | 0.285 | Numeric (log ug/L) |
